# A feasibility study of deep learning prediction model for VMAT patient-specific QA

**DOI:** 10.3389/fonc.2025.1509449

**Published:** 2025-03-26

**Authors:** Junjie Miao, Yuan Xu, Kuo Men, Jianrong Dai

**Affiliations:** Department of Radiation Oncology, National Cancer Center/National Clinical Research Center for Cancer/Cancer Hospital, Chinese Academy of Medical Sciences and Peking Union Medical College, Beijing, China

**Keywords:** gamma passing rate, deep learning, Monte Carlo, quality assurance, ArcCHECK

## Abstract

**Purpose:**

This study introduces a deep learning (DL) model that leverages doses calculated from both a treatment planning system (TPS) and independent dose verification software using Monte Carlo (MC) simulations, aiming to predict the gamma passing rate (GPR) in VMAT patient-specific QA more accurately.

**Materials and method:**

We utilized data from 710 clinical VMAT plans measured with an ArcCHECK phantom. These plans were recalculated on an ArcCHECK phantom image using Pinnacle TPS and MC algorithms, and the planar dose distributions corresponding to the detector element surfaces were utilized as input for the DL model. A convolutional neural network (CNN) comprising four layers was employed for model training. The model’s performance was evaluated through multiple predictive error metrics and receiver operator characteristic (ROC) curves for various gamma criteria.

**Results:**

The mean absolute errors (MAE) between measured GPR and predicted GPR are 1.1%, 1.9%, 1.7%, and 2.6% for the 3%/3mm, 3%/2mm, 2%/3mm, and 2%/2mm gamma criteria, respectively. The correlation coefficients between predicted GPR and measured GPR are 0.69, 0.72, 0.68, and 0.71 for each gamma criterion. The AUC (Area Under the Curve) values based on ROC curve for the four gamma criteria are 0.90, 0.92, 0.93, and 0.89, indicating high classification performance.

**Conclusion:**

This DL-based approach showcases significant potential in enhancing the efficiency and accuracy of VMAT patient-specific QA. This approach promises to be a useful tool for reducing the workload of patient-specific quality assurance.

## 1 Introduction

In the realm of modern radiation therapy, intensity-modulated radiation therapy (IMRT) and volumetric modulated arc therapy (VMAT) have become the most widely used techniques due to their superior dose conformity to the target volume (1). Even though these techniques are sophisticated, they are fundamentally complex and require patient-specific quality assurance (PSQA) to guarantee dose delivery accuracy (2). Traditionally, measurement-based quality assurance has been used to do the verification utilizing ionization chambers, films, or multi-dimensional detector arrays.

Gamma analysis, which examines the discrepancy between the measured and computed dose distributions, is commonly used in the verification process. For a given set of doses and distance-to-agreement (DTA) criteria, the gamma index is calculated using percentage dose difference and DTA at each detector. Traditional measurement-based methods, while effective, are labor-intensive and time-consuming, particularly when the initial results don’t meet acceptance criteria (3, 4). Traditional methods also impede the implementation of online adaptive radiotherapy, necessitating a rapid and real-time process for treatment planning and quality assurance (5, 6).

Recently, machine learning (ML) has been introduced for prediction of GPR. The complexity of IMRT and VMAT plans, characterized by factors such as the modulation complexity score (MCS), leaf motion constraints, and gantry speed etc., has been scrutinized for its impact on treatment accuracy and GPR (7–11). With ML, researchers have successfully harnessed these and treatment plan parameters to develop predictive models (12–21). These models are capable of directly forecasting the pass rate of radiation therapy plans, marking a significant advancement in PSQA. However, these models still face challenges. One issue is that these models often require manual selection of parameters, and inappropriate parameter choices can significantly affect prediction results.

Deep learning has been leveraged to enhance the accuracy and efficiency of predicting the GPR in treatment plans without defining various features like ML, otherwise extracting features from images automatically (22–25). Interian et al. and Tomori et al. both utilized convolutional neural networks to predict the GPR based on the 2D dose information (26, 27). Huang et al. introduced a virtual PSQA method for IMRT, employing UNet++ trained to forecast three key outputs (gamma pass rates, dose differences, and classification outcomes) to ascertain the success or failure of the QA process (28). Additionally, researchers have utilized fluence maps and 3D dose distributions to predict the verification pass rates or gamma distributions of radiation therapy plans (29–31). This kind of approaches involve analyzing the intricate patterns in dose distribution across three dimensions, providing a comprehensive view that enhances the accuracy of predicting treatment plan quality and effectiveness.

Independent calculation-based dose verification is another potential method recommended by the AAPM report TG219 (32). As a **Supplementary Method**, calculation-based dose verification is characterized by its low manpower consumption and high level of automation. However, it should be noted that this calculation method cannot replace the measurement-based patient-specific QA now. They serve as a supplementary check, particularly as certain machine delivery errors may not be detectable solely through software calculations (33, 34).

Moreover, the application of Monte Carlo methods, known for their high accuracy in dose calculation, has been integrated into predictive models (35–40). Monte Carlo methods provide highly accurate dose calculations by simulating the interaction of radiation with matter at the particle level. The method takes into account complex variables such as tissue heterogeneity, irregular geometries, and detailed dose deposition patterns. Additionally, Monte Carlo simulations can model the probabilistic nature of radiation transport, allowing for more precise dose distributions compared to traditional deterministic methods. These factors make the Monte Carlo methods suitable for PSQA. Some third-party radiotherapy treatment plan verification software, such as SunCHECK, Mobius3D, ArcherQA, and RadCalc, complement plan verification efforts and play an important role in ensuring the precision and safety of radiation therapy. These software applications commonly employ the highly accurate Monte Carlo-based dose calculation methods for dose computation. The combination of advanced dose calculation algorithms with AI methodologies offers a promising avenue for enhancing the precision and efficiency of plan verification processes.

In this context, the purpose of this study is to investigate the feasibility and performance of DL integrated with doses derived from TPS and independent verification software using Monte Carlo dose calculations in the PSQA. This approach is expected to offer a novel tool for ensuring the accuracy and safety of treatments.

## 2 Materials and methods

### 2.1 Study design

**Figure 1:**
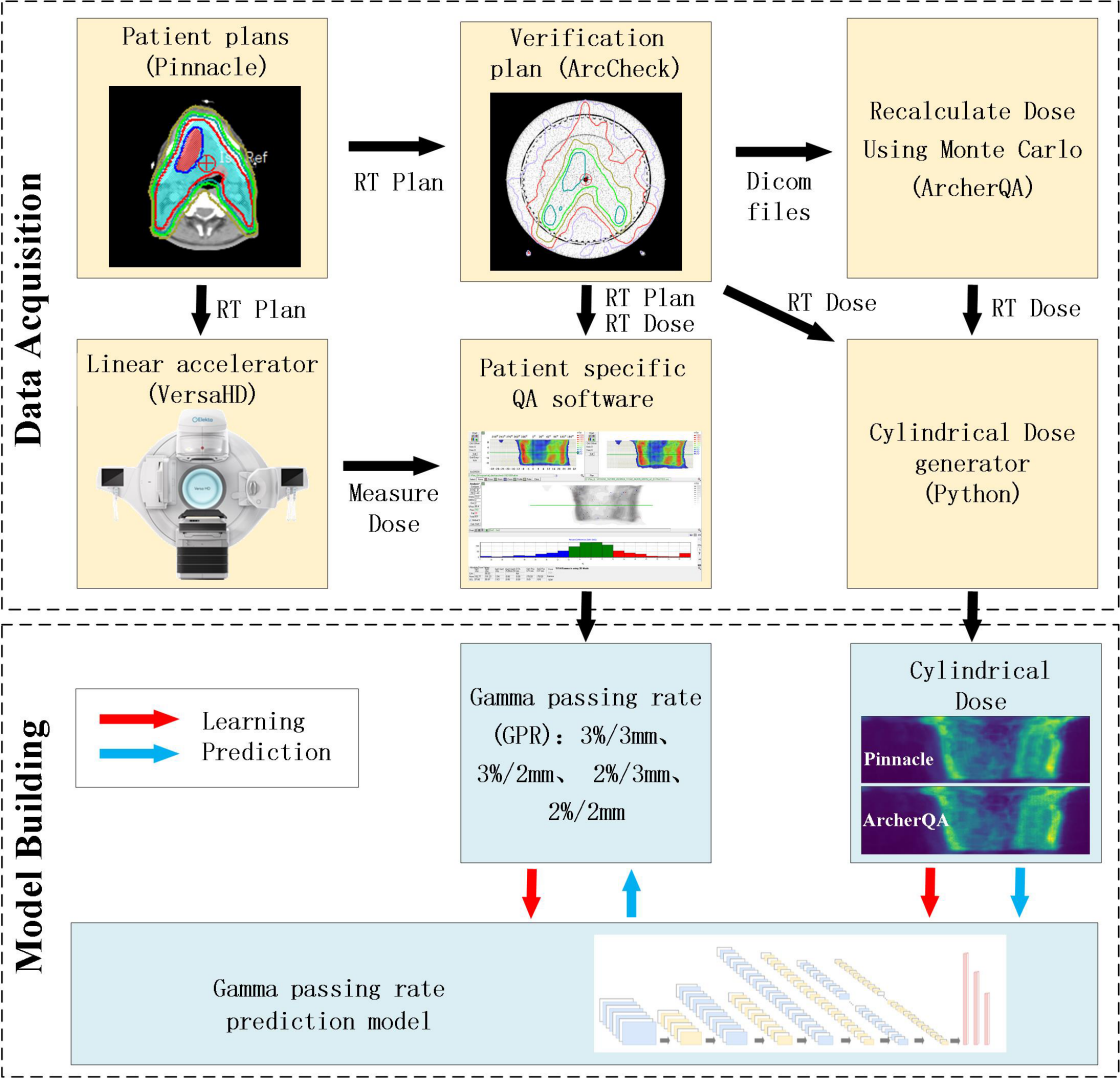
The flowchart for the study design.

The overall study design for predicting GPR is shown in **Figure 1**. Initially, patient plans were optimized within the TPS. Subsequently, the RT plan was transferred to the accelerator for dose measurement. Doses were recalculated for the clinical plans on a QA phantom using both the TPS and plan verification software ArcherQA (Wisdom Technology Company Limited, Hefei, China) with the MC algorithm. Gamma analysis was performed by comparing the TPS-calculated dose on the QA phantom against the measured dose, obtaining GPR with different criteria. A Python script was utilized to extract cylindrical dose data corresponding to the detector array from the TPS and ArcherQA exports. The cylindrical dose, along with the GPR were utilized for training convolutional neural network models and predictive analysis.

### 2.2 Clinical equipment and data acquisition

We retrospectively selected 710 clinical VMAT plans treated with a 6-MV photon beam delivered by an Elekta Versa HD accelerator (Elekta AB, Stockholm, Sweden) in flattering filter-free mode. The treatment sites encompassed the head and neck (209), thorax (198), abdomen (205), and pelvis (98). The distribution of disease sites across the training, validation, and testing datasets is detailed in **Supplementary Appendix A**. For these plans, the VMAT optimization was carried out using Pinnacle TPS (version 16.2, Philips Healthcare, Eindhoven, Netherlands). Within this system, the dose calculation was performed using an adaptive convolve dose engine.

The ArcCHECK dosimetry system (Sun Nuclear Corporation, Melbourne, USA) was used to perform verification measurements. ArcCHECK is a helical 3D detector array comprising 1386 diodes arranged within the cylindrical wall of a phantom. Each patient plan was recalculated on ArcCHECK with a dose grid resolution of 0.2 cm in each dimension. The GPR was calculated with SNC patient software (version 6.7.2, Sun Nuclear Corporation, Melbourne, USA) for evaluating dose discrepancies between the TPS-calculated and phantom-measured values. For the gamma analysis (3D mode), we applied criteria of 3%/3 mm, 3%/2 mm, 2%/3 mm, and 2%/2 mm, alongside a 10% dose threshold, using absolute dose mode and global normalization (41, 42).

All the DICOM files of verification plans, including the RT plan, RT structure, RT dose, and CT image, were imported into ArcherQA to recalculate the dose distribution using the Monte Carlo algorithm. ArcherQA has already been clinically implemented for all our machines for independent dose verification. The details for beam modeling was introduced before (43). The modeling was also commissioned and validated with phantom measurement results. The uncertainty was 1% for transportation used in Monte Carlo simulations. The grid for Monte Carlo calculation were identical to the settings in the validation plan within the TPS.

The calculated 3D dose was exported from the TPS and ArcherQA, respectively, to our in-house cylindrical dose generator. This Python-based tool extracts the dose distribution on the cylindrical surface where the detector array resides. The extracted dose distribution, with a resolution of 220×673, has been normalized to the maximum dose value. Finally, the dose distribution data is saved in the form of an h5 file, which is then used for the training and testing of the model.

### 2.3 Model architecture and training parameters

**Figure 2:**
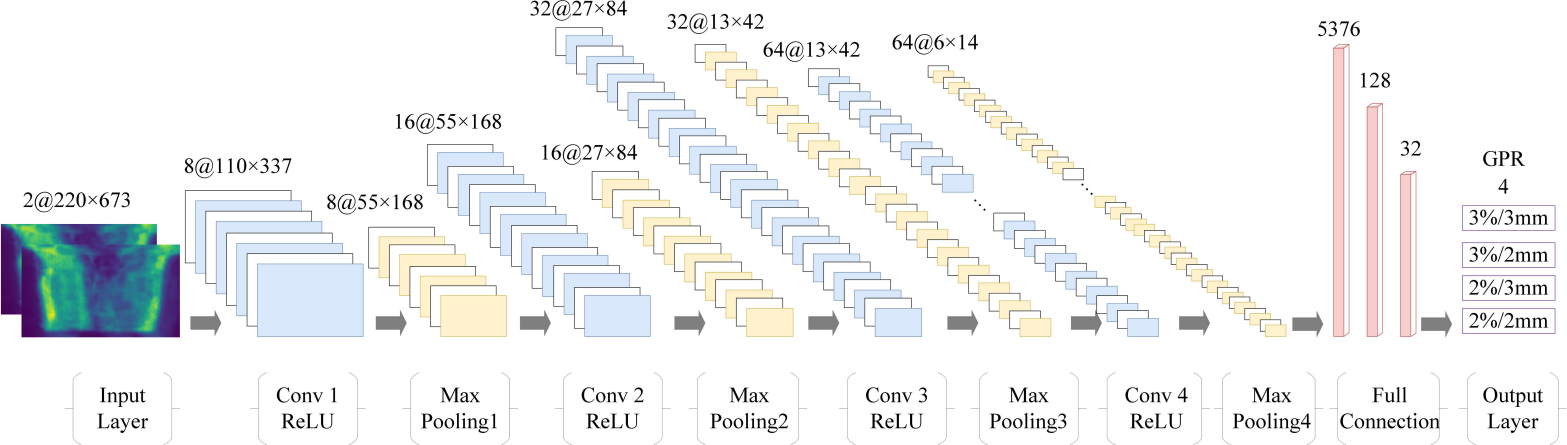
Framework and model architecture for PSQA prediction based on CNN.

In this study, a convolutional neural network (CNN) was used to predict the GPR. **Figure 2** illustrates the model’s architecture, which comprises four convolutional layers, four max-pooling layers, four activation layers, a flatten layer, and three fully connected layer. All activation layers utilized the rectified linear unit (ReLU) function. ReLU helps improve the efficiency of learning by eliminating output values below zero. Additionally, dropout layer with a 0.25 drop rate before the first fully connected layer was employed to enhance network robustness, mitigating overfitting by randomly removing neurons (44). The model’s input consists of dual-channel unwrapped dose on the cylindrical surface, with doses originating from both the TPS and ArcherQA. The model’s output is gamma pass rates for four different criteria.

Our PSQA dataset consisted of 710 case records. For the dataset splitting, we allocated 426 cases for training, 142 for validation, and the remaining 142 for testing. During model training, we used data augmentation strategies, such as translations and flipping, to artificially increase the size and diversity of the dataset. The neural network architecture is based on PyTorch and was trained on an NVIDIA GeForce RTX 4080 GPU. During training, the L1 Loss (Mean Absolute Error) was used as our loss function. The optimization process utilized the Adam optimizer with a base learning rate (lr) set to 0.0001 and adaptive moment estimation parameters (betas) configured as (0.9, 0.999). To enhance training dynamics, a learning rate scheduler was employed, gradually reducing the learning rate by a factor of 0.98 every 5 epochs. The deep learning models were trained for a maximum of 300 epochs. Early stopping was employed to prevent overfitting by halting training when the validation loss started to increase.

### 2.4 Evaluation

In pursuit of a dependable and consistent model, a 5-fold cross-validation approach was used to train the model. Through cross-validation, optimal model parameters were determined and then used to generate predicted GPR for the 142 test cases. Subsequently, a GPR comparison was performed between the predicted GPR and the measured GPR, taking into account metrics of mean error (ME), mean absolute error (MAE), and root-mean-square error (RMSE) as described by Equations 1–3.


(1) $$ME=\frac{1}{N}\sum_{i=1}^{N}\left(m\left(i\right)-p\left(i\right)\right)$$

(2) $$MAE=\frac{1}{N}\sum_{i=1}^{N}\left|m\left(i\right)-p\left(i\right)\right|$$

(3) $$RMSE=\sqrt{\sum_{i=1}^{N}\frac{{\left(m\left(i\right)-p\left(i\right)\right)}^{2}}{N}}$$

where *N* represents the total count of test cases, *p(i)* denotes the GPR value predicted for the *i*-th case, and *m(i)* signifies the corresponding measured GPR value.

The performance across various criteria was also evaluated using the ROC curve. This curve represents the relationship between the true positive rate (TPR) and the false positive rate (FPR), as described by Equations 4, 5. To quantify the classifier’s performance, the AUC (Area Under the Curve) was calculated. Typically, AUC values range from 0.5 to 1, with 0.5 indicating random classification, and a value close to one signifying an excellent classifier.


(4) $$TPR=\frac{N\left(TP\right)}{N\left(TP+FN\right)}$$

(5) $$FPR=\frac{N\left(FP\right)}{N\left(FP+TN\right)}$$

where, *N* represents the number of a specific value, and definitions for TP (true positive), TN (true negative), FP (false positive), FN (false negative) are provided in **Table 1**. The “pass” threshold was defined as achieving a GPR greater than or equal to 95% at 3%/3 mm, greater than or equal to 90% at 3%/2 mm, 2%/3 mm, and 2%/2 mm (41, 42). Otherwise, it was classified as “fail”.

**Table 1:** Definitions of categories for ROC analysis.

Category	Predicted GPR	Measured GPR
TP	Fail	Fail
TN	Pass	Pass
FP	Fail	Pass
FN	Pass	Fail

TP, true positive; TN, true negative; FP, false positive; FN, false negative.

## 3 Results

**Figure 3:**
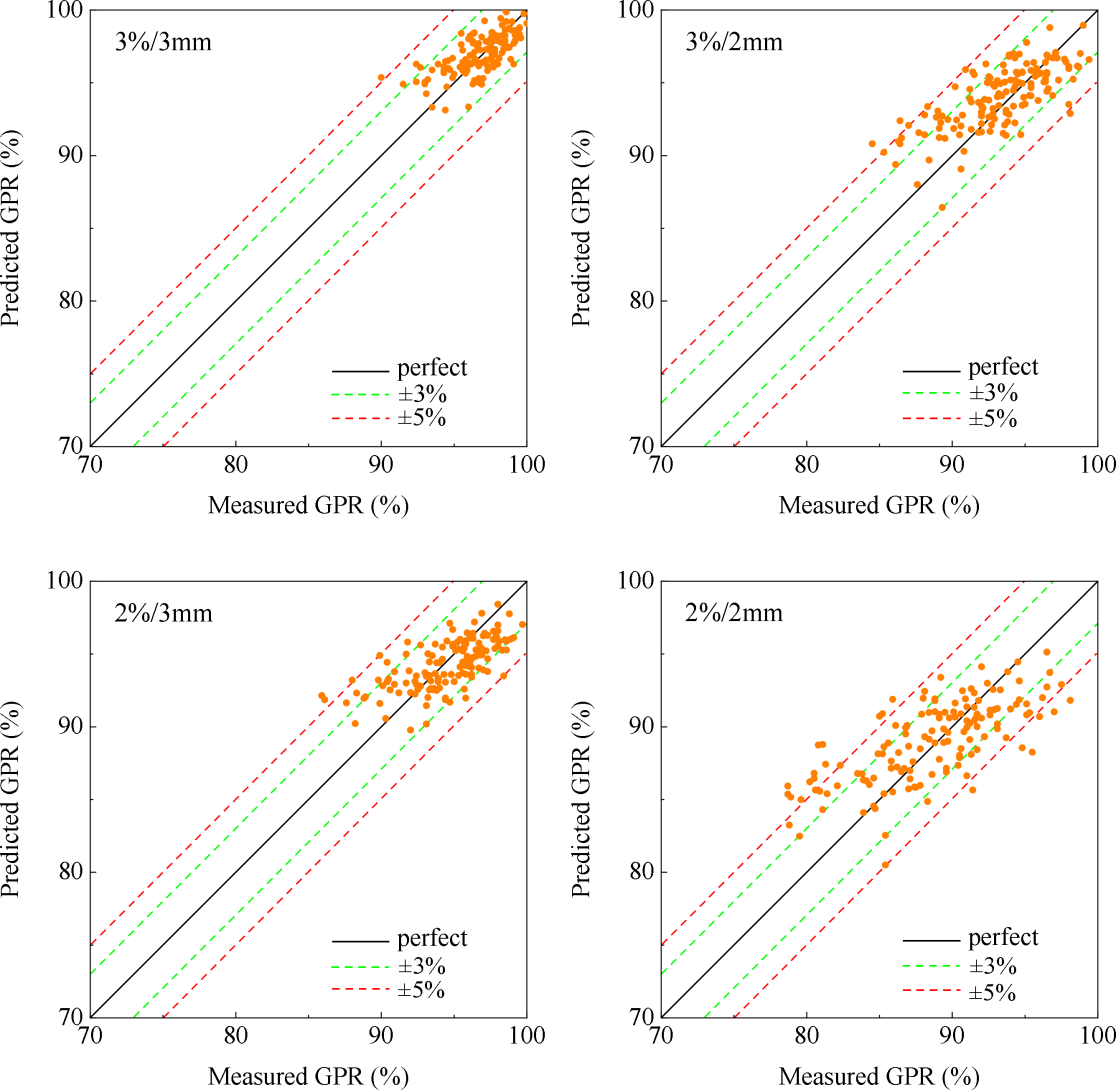
Plot of measured and predicted GPR at 3%/3 mm criterion, 3%/2 mm criterion, 2%/3 mm criterion, and 2%/2 mm criterion.

The entire training process for the model takes around 110 minutes, and the detailed training and validation loss curves over epochs can be found in **Supplementary Appendix B**. **Figure 3** illustrates the correlation between measured and predicted GPR values for the 3%/3mm, 3%/2mm, 2%/3mm, and 2%/2mm gamma criteria. The black diagonal line in the figure represents a perfect prediction where the measured values equal the predicted values. **Table 2** presents mean values and standard deviations (SD) for both measured and predicted GPR values of the test cases.

**Table 2:** Measured and predicted GPR values (%) of test cases for various criteria.

Criterion	Measured GPR		Predicted GPR	
	Mean	SD	Mean	SD
3%/3mm	96.9	1.9	97.1	1.4
3%/2mm	93.3	3.2	94.0	2.2
2%/3mm	94.6	2.8	94.3	1.7
2%/2mm	88.6	4.6	89.0	2.8

SD, standard deviation.

**Figure 4:**
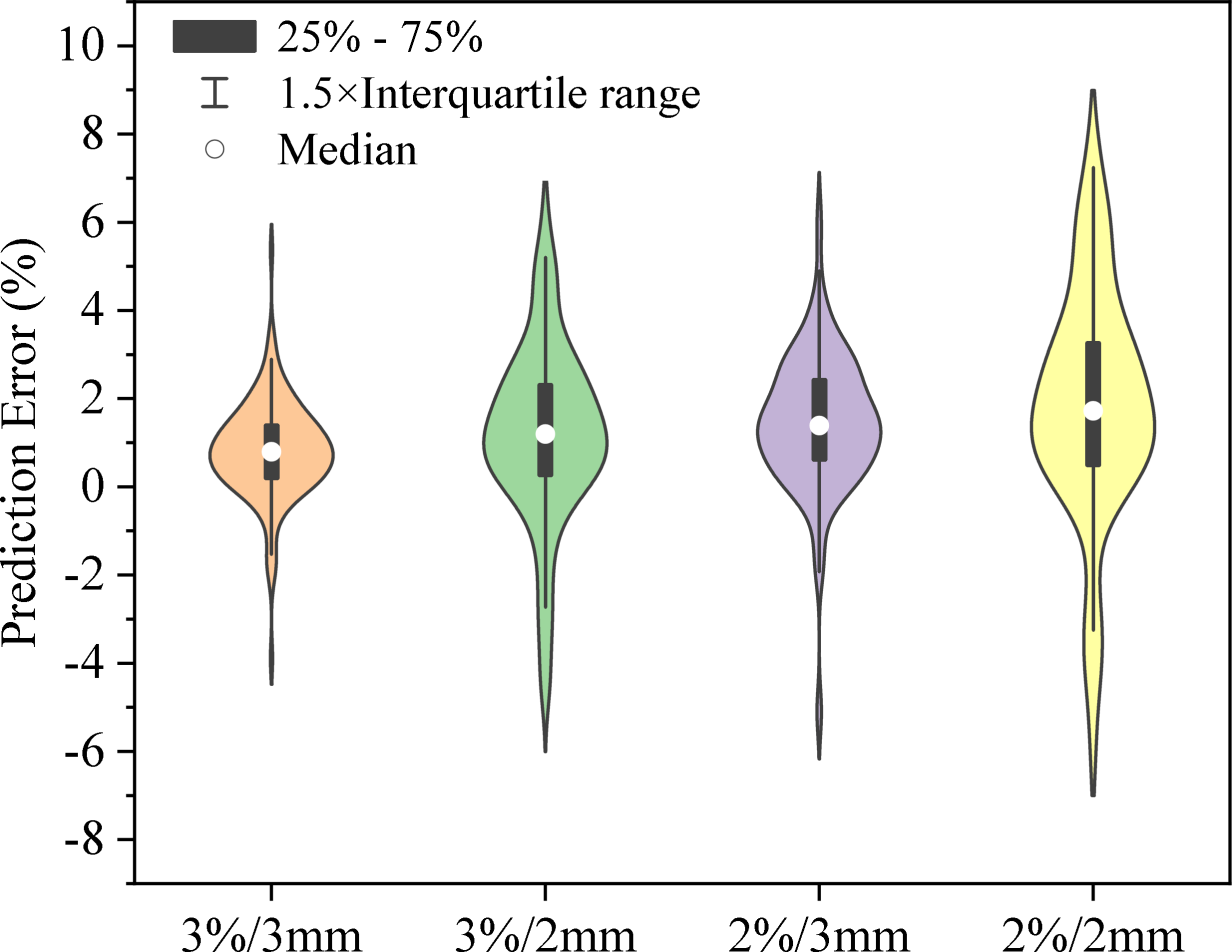
The prediction error distribution for GPR values across four different criteria: 3%/3mm, 3%/2mm, 2%/3mm, and 2%/2mm. The black bars represent the interquartile range (25% - 75%), the lines extending from the bars indicate the 1.5× interquartile range, and the white circles show the median prediction error for each category. The shape of each violin plot illustrates the probability density of the data at different error levels, with wider sections representing a higher probability of data points falling at a particular error percentage.

**Figure 4** shows the distribution of prediction errors for GPR values across four distinct criteria: 3%/3mm, 3%/2mm, 2%/3mm, and 2%/2mm. Specific statistical error metrics such as ME, MAE, and RMSE can be found in **Tables 3** and **4**. The Pearson’s correlation coefficient (CC) between predicted GPR and measured GPR was calculated to be 0.69, 0.72, 0.68, and 0.71 for the 3%/3mm, 3%/2mm, 2%/3mm, and 2%/2mm criteria, respectively. The results demonstrate a robust correlation between the predicted and measured gamma pass rates.

**Table 3:** ME, MAE, RMSE, and CC computed from measured and predicted GPR (%).

Criterion	ME	MAE	RMSE	CC
3%/3mm	-0.2	1.1	1.4	0.69
3%/2mm	-0.8	1.9	2.4	0.72
2%/3mm	0.3	1.7	2.1	0.68
2%/2mm	-0.4	2.6	3.3	0.71

ME, mean error; MAE, mean absolute error; RMSE, root mean squared error; CC, Pearson’s correlation coefficient.

**Table 4:** Comparison of GPR (%) error statistics in the test dataset among different model inputs under various gamma criteria.

Criterion	TPS&MC			TPS Only			MC Only		
	ME	MAE	RMSE	ME	MAE	RMSE	ME	MAE	RMSE
3%/3mm	-0.2	1.1	1.4	-0.4	1.4	1.8	0.6	1.5	1.9
3%/2mm	-0.8	1.9	2.4	-1.1	2.2	2.7	1.0	2.5	3.1
2%/3mm	0.3	1.7	2.1	-0.6	1.9	2.5	-0.9	2.0	2.7
2%/2mm	-0.4	2.6	3.3	0.7	3.0	3.8	-1.1	3.2	4.1

TPS & MC, TPS Only, and MC Only represent the different dose configurations used as inputs for the predictive model.

**Figure 5:**
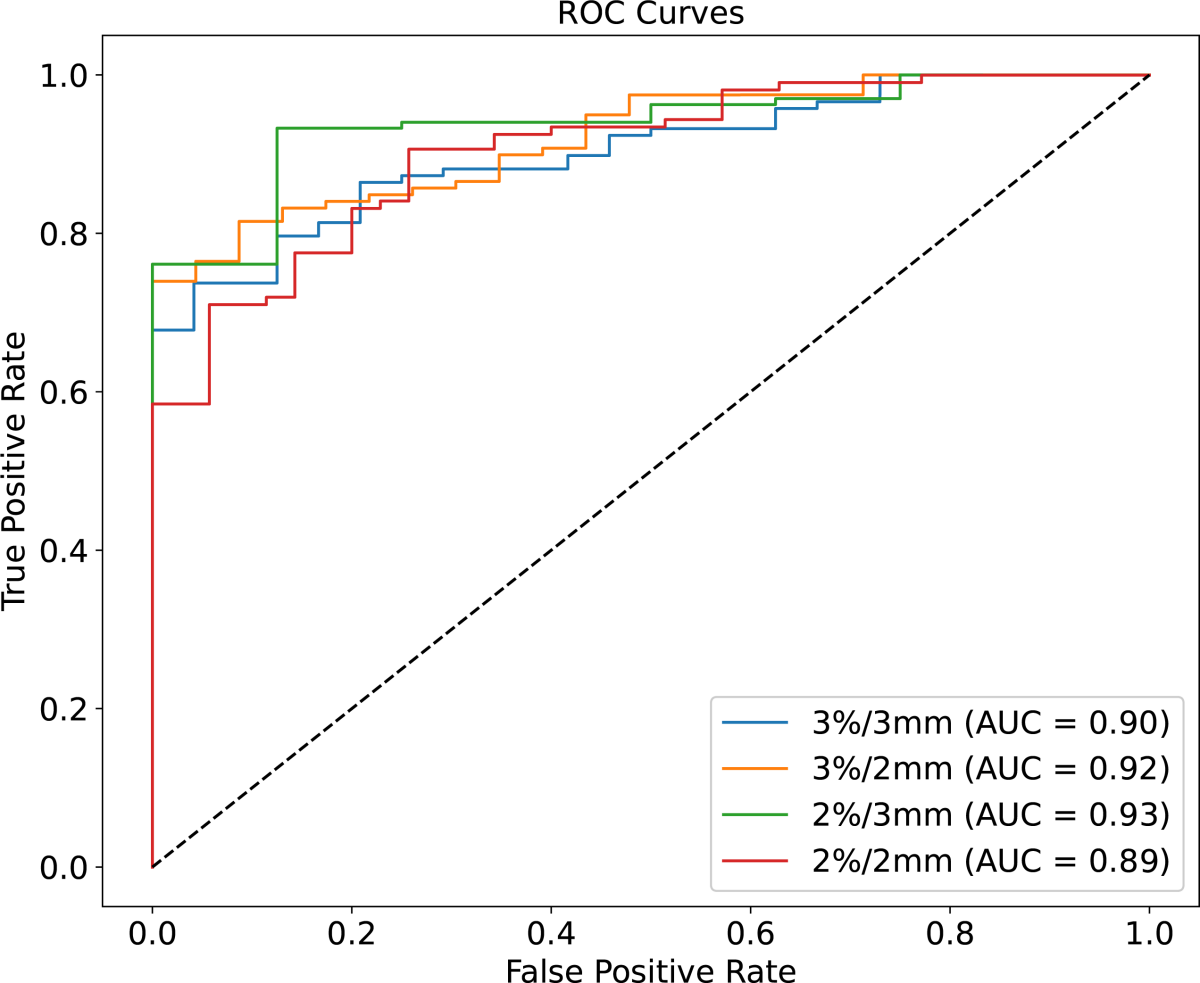
ROC curves for four different criteria of predicted GPR. Each curve represents the trade-off between the true positive rate (sensitivity) and false positive rate (1-specificity) for the 3%/3mm (blue line, AUC = 0.90), 3%/2mm (orange line, AUC = 0.92), 2%/3mm (green line, AUC = 0.93), and 2%/2mm (red line, AUC = 0.89) criteria.

The ROC curves for different GPR value classification criteria are illustrated in **Figure 5**, with the AUC values being 0.90, 0.92, 0.93, and 0.95 for the 3%/3mm, 3%/2mm, 2%/3mm, and 2%/2mm criteria, respectively. The ROC curve and the corresponding AUC values serve as key indicators of the model’s classification performance. An AUC value closer to 1 indicates excellent predictive accuracy, suggesting that the model is effectively distinguishing between true positive and true negative cases across the different criteria. These AUC values indicate that each criterion provides excellent predictive accuracy and high classification performance.

## 4 Discussion

In this study, we present an exploration of PSQA using DL for predicting GPR in radiation therapy. Traditional PSQA, primarily relies on measurement-based methods, which, although accurate, can be time-consuming and resource-intensive. The model developed in this study can rapidly provide planners with GPR results, allowing for timely actions like replanning when predicted GPR falls below acceptance criteria. High-accuracy predictions enhance the credibility of the virtual QA method and support decision-making. Our prediction model can be integrated into an automated software platform. When new plan data is received, the platform automatically extracts the dual-channel dose, calls the model for prediction, and generates a report. Compared to independent Monte Carlo calculations, this process only requires an additional few minutes of time, which fully meets the clinical application time requirements.

The International Commission on Radiation Units and Measurements (ICRU) Report 83 (45) and the American Association of Physicists in Medicine (AAPM) Task Group 219 Report (32) endorse the use of independent dose calculation methods as an additional verification step for treatment plans generated by TPS. Utilizing random sampling to simulate particle transport and interactions, the MC method not only ensures a more accurate representation of radiation transport and scattering in various media but also excels in dose calculations for small field sizes where traditional convolution methods may falter in precision. Consequently, these attributes have made MC algorithms commonly employed as independent dose calculation methods. Motivated by this, we explored the integration of advanced MC dose calculation algorithms and AI methodologies to achieve more accurate predictions of GPR. In this study, a commercial MC-based dose engine (ArcherQA) was used for independent dose calculations. In the model construction, we utilized a dual-channel dose as input, incorporating dose distributions from both the TPS and ArcherQA, to enhance the model’s performance. The model with dual-channel dose input exhibits smaller MAE and RMSE, as detailed in **Table 4** for specific comparative results. One possible reason is that the doses calculated by Monte Carlo algorithms are closer to the measured values, especially in the calculation of small field doses (39). However, due to the inherent lack of interpretability common in deep learning algorithms, a direct and reliable cause still requires further exploration.

GPR prediction has been explored by various researchers in prior studies. Notably, the AI-driven regression method exhibited favorable predictive performance. Hirashima et al. utilized plan complexity and dosiomics features as input data to predict the GPR value of a helical diode array, achieving correlation coefficients ranging from 0.45 to 0.61 and MAE ranging from 2.7% to 3.2% for a 3%/2mm gamma criteria (17). Matsuura et al. utilized deep learning to predict the GPR of a 3D detector array-based quality assurance for volumetric modulated arc therapy in prostate cancer (30). They achieved a correlation coefficient of 0.7 and a MAE of 2.5% for a gamma criterion of 3%/2mm. In the course of this study, we acquired a correlation coefficient of 0.72 and a MAE of 1.9% using the predictive model under a 3%/2mm gamma criterion. While direct comparisons of these results are challenging due to variations in prediction methods, such as treatment site and measurement device, our study demonstrated the DL model utilizing dual-channel dose input for GPR prediction outperforms or equals other ML or DL-based algorithms.

In the process of radiotherapy, many factors can lead to dose deviations. Our DL model is adept at detecting systemic dose calculation errors and discrepancies in fluence/geometric modeling of the treatment machine. It can also identify dose deviations resulting from suboptimal field sizes, such as those that are too small, too large, or significantly off-center. However, it does not track errors in data transfer to the treatment unit nor detect actual errors in treatment delivery if the machine fails to interlock itself, as these would require real-time monitoring or separate verification systems.

In spite of its efficacy, this approach faces certain challenges. Firstly, the model’s performance is inherently tied to the quality and diversity of the training data. Although our dataset encompasses multiple sites, it is limited to 710 clinical VMAT plans from a single machine, requiring further investigation into the model’s generalizability across different machines, treatment modalities, and patient populations. Before clinical implementation, the model requires training on a significantly larger dataset to ensure its robustness and reliability. During the initial phase of clinical use, the model’s predictions will be used in parallel with existing clinical methods to cross-validate results and ensure consistency. Another consideration is the model’s dependency on the accuracy of input data from TPS, ArcherQA and ArcCHECK. Any discrepancies or errors in these systems could negatively impact the model’s predictions. Therefore, continuous verification of these systems is essential if the model is to be applied in clinical settings.

Our approach offers several key advantages compared to similar techniques. Firstly, compared to machine learning-based methods, our method does not rely on the manual extraction of feature parameters, which reduces human bias and the risk of errors in feature selection. In comparison to third-party verification methods based solely on Monte Carlo simulations, our approach offers higher reliability. By incorporating dual-channel dose into the predictive model, it provides a more comprehensive assessment of the treatment plan’s quality. Furthermore, when compared to other deep learning-based models, our approach has demonstrated higher accuracy in predicting GPR, making it a more reliable tool for PSQA.

Our model introduces an innovative method to support and streamline the PSQA process. In the near future, if our model is applied clinically, it may first be used to assist in checking or screening low pass-rate verification plans. It would serve as a supplement rather than a replacement for traditional PSQA. The potential application of our deep learning model is to identify treatment plans with low gamma passing rates. This selective focus would enable clinical physicists to concentrate their efforts on plans that are most likely to require adjustments. Additionally, integrating this method with emerging technologies like magnetic resonance imaging-guided radiation therapy could provide novel insights into online adaptive radiation therapy strategies.

## 5 Conclusion

We proposed a deep learning-based prediction model for VMAT patient-specific QA, using extracted doses from both TPS and MC calculations as input to the model. The dual-channel dose distribution, associated with cylindrically arranged detector elements, captures plan characteristics and holds predictive potential for the GPR in VMAT plans. This method is expected to be a valuable tool capable of reducing the workload associated with PSQA.

## Data Availability

The raw data supporting the conclusions of this article will be made available by the authors, without undue reservation.
