# Supplementary material for: A feasibility study of deep learning prediction model for VMAT patient-specific QA
Source: Front Oncol. 2025 Mar 26;15:1509449. doi: 10.3389/fonc.2025.1509449 (PMC11979141; doi:10.3389/fonc.2025.1509449)

**Supplementary Materials**

**Appendix A:** **The details of disease sites spread out amongst the training, validation and testing datasets.**

There are 710 plans in total, with 209 plans for Head and Neck, 198 plans for Thorax, 205 plans for Abdomen, and 98 plans for Pelvis. For specific details, see the table below.

|  | Training | Validation | Testing | Total |
| --- | --- | --- | --- | --- |
| Head and neck | 125 | 42 | 42 | 209 |
| Thorax | 118 | 40 | 40 | 198 |
| Abdomen | 123 | 41 | 41 | 205 |
| Pelvis | 58 | 20 | 20 | 98 |

**Appendix B: Training and validation loss curves (L1) over epochs.**

The black line represents the training loss, while the orange line shows the validation loss.


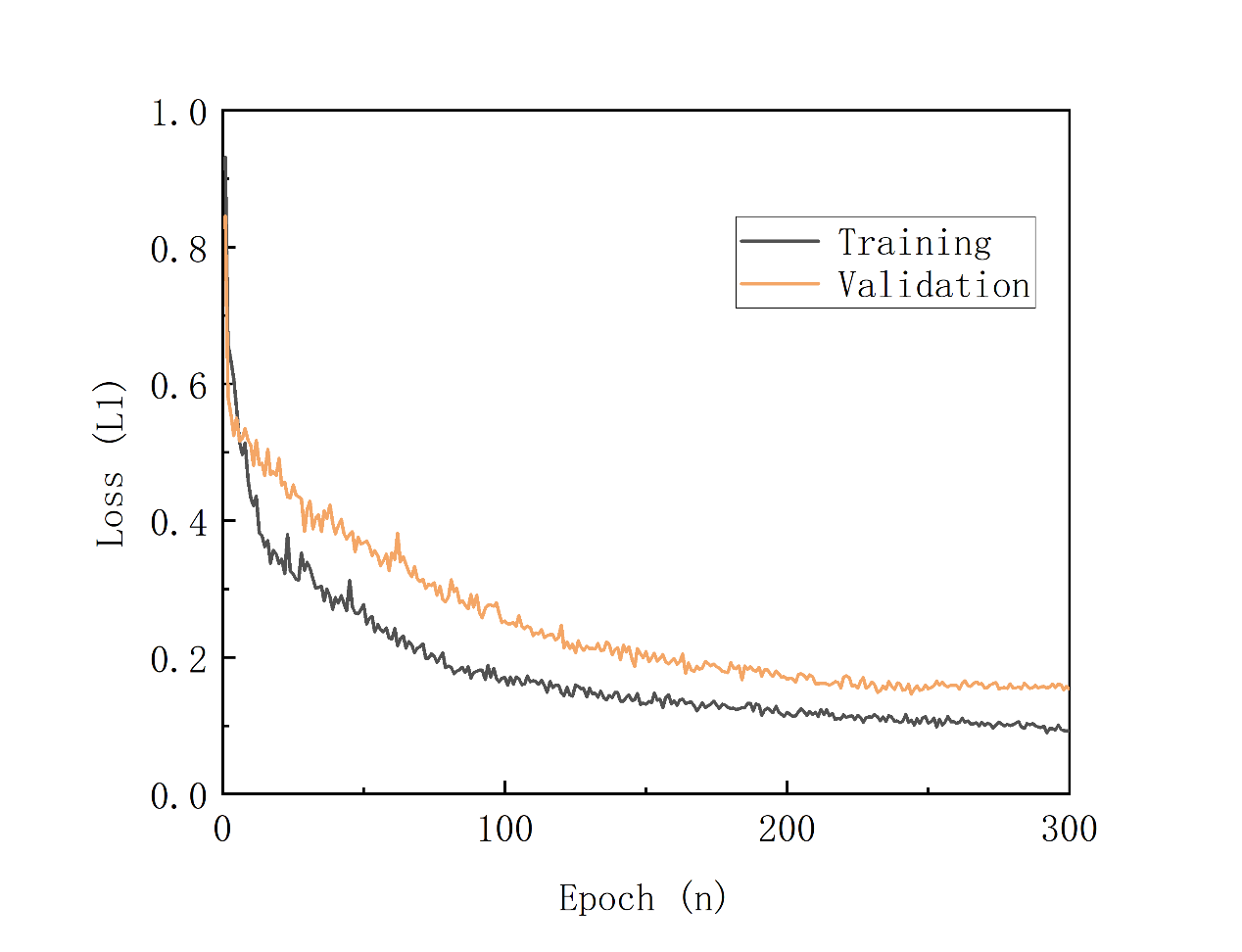

Supplement: Supplementary file 1 [file DataSheet1.docx]
